# Vaa3D-x for cross-platform teravoxel-scale immersive exploration of multidimensional image data

**DOI:** 10.1093/bioinformatics/btac794

**Published:** 2023-01-05

**Authors:** Jiangshan Liang, Luchen Deng, Shize Chen, Yimin Wang, Zongcai Ruan, Lingli Zhang

**Affiliations:** School of Computer Science and Engineering, Southeast University, Nanjing, Jiangsu, China; School of Computer Science and Engineering, Southeast University, Nanjing, Jiangsu, China; School of Computer Science and Engineering, Southeast University, Nanjing, Jiangsu, China; Guangdong Institute of Intelligence Science and Technology, Hengqin, Guangdong, China; School of Computer Science and Engineering, Southeast University, Nanjing, Jiangsu, China; School of Biological Science and Medical Engineering, Southeast University, Nanjing, Jiangsu, China

## Abstract

**Summary:**

Vaa3D is a software package that has been widely used to visualize and analyze multidimensional microscopic images in a number of cutting edge bioimage informatics applications. However, due to many recent updates of both software development environments and operating systems, it was highly requested to maintain Vaa3D and disseminate it on latest operating systems. In addition, there has never been a showcase about how to use Vaa3D’s cross-platform visualization and immersive exploration functions for multidimensional and teravoxel-scale images. Here, we introduce a newly developed version of the software, called Vaa3D-x, to address all the above issues.

**Availability and implementation:**

Vaa3D-x is released in both binary and Open-Source available at vaa3d.org and GitHub (https://github.com/Vaa3D).

**Supplementary information:**

Supplementary data are available at *Bioinformatics* online.

## 1 Introduction

Current high-throughput microscopy techniques enable generation of a massive amount of multidimensional imaging data, which are often at the scale of teravoxel or even peta-voxel. As a result, there has been a very strong demand to develop tools to fulfill the emerging need of dealing and analyzing such massive image datasets. In the Open-Source world, for over a decade Vaa3D has been a widely adopted platform software package for multidimensional visualization and exploration (interaction, annotation, processing, etc) of very large multidimensional volume (Peng *et al.*, 2010; 2014; Wang *et al.*, 2019). Vaa3D is cross-platform, supports real-time 3D visualization and analysis capabilities to images of potentially unlimited size (Vaa3D-terafly) and has almost 500 plugins for image acquisition, data management, image processing, data analysis and pipelining. Compared with other bioimage analysis tools (Jeong *et al.*, 2010; Long *et al.*, 2012; Pietzsch *et al.*, 2015; Schroeder *et al.*, 2021), Vaa3D stands out with its intrinsic design to handle large, hierarchically organized multidimensional data volumes and associated surface objects. These features make Vaa3D a natural choice in many large-scale studies that involve hundreds or thousands of multidimensional images.

Vaa3D was originally developed based on Qt4 using C++. Due to multiple recent updates of Qt libraries and operating systems (OS) that are no longer compatible with each other, there is a strong need to re-develop several core parts of Vaa3D so it can be more easily disseminated on latest operating systems. On the other hand, as many users of Vaa3D are using the Windows OS, for which the previous Vaa3D software was built mostly with the currently obsolete Visual Studio 2013 compiler, it is needed to also upgrade the key building scripts of Vaa3D to be OS-independent to simplify the maintenance.

In this work, we developed Vaa3D-x by providing a comprehensive solution that does not only update development environment to maintain and disseminate the software more easily but also allow cross-platform users to visualize and annotate teravoxel-scale images using both hierarchical image annotation and immersive virtual reality. We believe these features can help a broad user group to tackle their big imaging data more efficiently.

## 2 Application

**Fig. 1.**
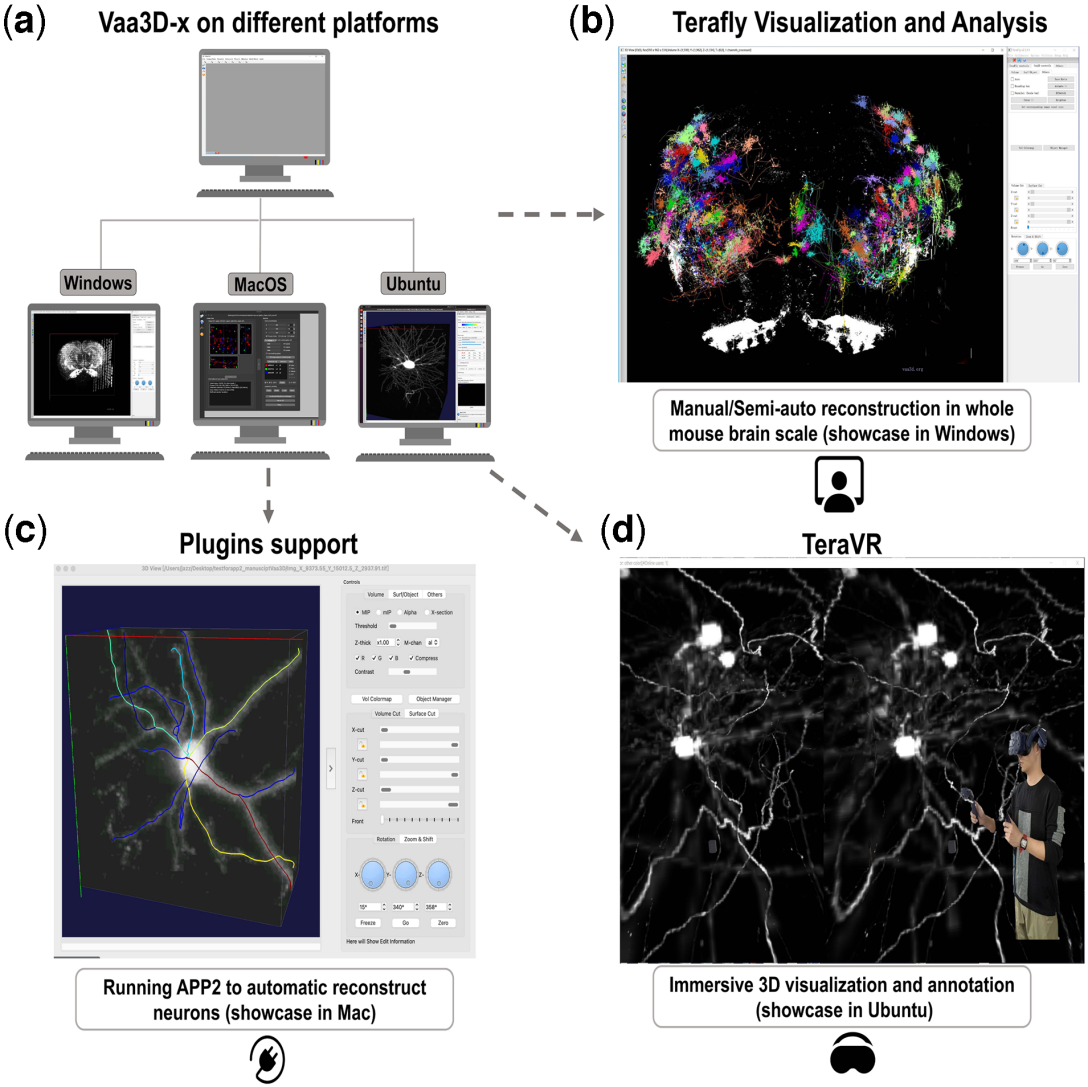
Vaa3D-x cross-platform functions. (**a**) Vaa3D-x can be used for visualizing and exploring large-scale data in three platforms (Windows, Linux and Mac). (**b**) TeraFly function for manual/semi-auto reconstructing in whole mouse brain scale on Windows. (**c**) An example of running APP2 plugin on Mac. (**d**) An example of using TeraVR for immersive 3D visualization and annotation on Linux (Ubuntu)

We applied Vaa3D-x to quantitative visualization and analysis of teravoxel-scale multidimensional images on all major OS platforms, including Windows, Linux and Mac. We used TeraFly module of Vaa3D-x for real-time hierarchical visualization of whole brain imaging data (Fig. 1), which has several dozens of teravoxels. In addition, we also upgraded the TeraVR module, which was the first teravoxel-scale immersive visualization tool in the field. Currently, TeraVR does not run on Mac due to the Apple company’s limited support on specific virtual reality hardware. We also upgraded more than 100 plugins in Vaa3D-x, specifically for image processing such as image filtering, segmentation, registration as well as neuron morphology tracing and analysis. We also performed tests to illustrate the efficiency and robustness of Vaa3D-x (Supplementary Fig. S2) and a thorough functional testing plan for Vaa3D-x across multiple platforms and configurations to increase the confidence level of the performance of the software (Supplementary Tables S2–S4).

Figure 1 shows a typical workflow to use Vaa3D-x in the application of neuron tracing from whole brain images where neurons are labeled fluorescently. In this example, one Vaa3D-x plugin for automatic tracing, APP2, is used to produce 3D reconstruction of a neuron quickly, followed by manual curation to annotate the neuron using the TeraFly module. Further, the TeraVR module can be used to annotate and correct the reconstruction error of the 3D morphology of neurons in the virtual reality space. Hundreds of neuron dendrites can also be visualized all together when needed (Fig. 1b).

## 3 Method

We unified the compiler (g++) on whole platform and removed outdated compiler (Visual Studio 2013) on which Vaa3D relied. For the development system, we used Qt6 and abandoned Qt4 which caused the compilation conflict on Mac OS system. What’s more, all Vaa3D external dependency libraries were recompiled and stored in advance according to the system, which transformed the compilation process from script compilation to one-click compilation relying on Qt Creator (Supplementary Fig. S1). The OpenVR library was also successfully deployed on Windows and Ubuntu20, which meant that TeraVR can bring immersive annotations and analysis to users on the above platform. We discarded the original SDL window and instead embraced SteamVR with its own viewport.

Vaa3D-x optimized the source code systematically and implemented new interfaces instead of these functions deprecated by Qt itself. Meanwhile, more C++17 features were applied in TeraFly to keep the code advanced, and conflicts were all emendated to make the previous functions workable and compatible in new Qt6 environment. Many new features were also added.

## 4 Conclusion

Compared to the old version of Vaa3D, the unique differences of Vaa3D-x are: The key building scripts of the software have been upgraded, the outdated Visual Studio 2013 compiler has been obsoleted, and the development system has been upgraded from Qt4 to Qt6. These changes made Vaa3D-x be OS-independent and more easily disseminated on latest operating systems. The upgraded modules Terafly and TeraVR in Vaa3D-x allow cross-platform users visualize and immersive explore functions for multidimensional and teravoxel-scale images using both hierarchical image annotation and immersive virtual reality. The compilation of Vaa3D-x has also been simplified, which can help a broader user group.

## Supplementary Material

btac794_Supplementary_Data

## Data Availability

The data underlying this article are available in Vaa3D / Vaa3D_Data Repository, at https://github.com/Vaa3D/Vaa3D_Data/releases/tag/data_v2.0.
