# Supplementary material for: Vaa3D-x for cross-platform teravoxel-scale immersive exploration of multidimensional image data
Source: Bioinformatics. 2023 Jan 5;39(1):btac794. doi: 10.1093/bioinformatics/btac794 (PMC9832945; doi:10.1093/bioinformatics/btac794)
Supplement: btac794_Supplementary_Data [file btac794_supplementary_data.zip › Supplementary Material_Revised(clean).pdf]

Vaa3D-x: Supplementary information

Supporting material for the publication “Vaa3D-x for Cross-platform Teravoxel-scale Immersive Exploration of Multidimensional Image Data”

1 Software Installation

(1) Go to Release Vaa3D-x Release 1.1.2 · Vaa3D/release (github.com) ,download the Vaa3D-x version.

(2) Choose the appropriate program corresponding to your operating system (Mac, Linux or Windows) and download it to your local computer.

(3) Run the Vaa3D-x program to launch the GUI.

- For Windows, double-click the program Vaa3D-x.exe.
- For Linux, enter the folder that contains the unzipped program and start Vaa3D-x.sh command to run.
- For Mac, double-click the program Vaa3D-x.app or on the command line console run the command Contents/MacOS/Vaa3D-x.app to start the GUI with the running log information displayed. Due to Mac limitations, using Vaa3D for the first time requires calling sudo spctl --master-disable on the command line, and then allowing developers from any source in general.

2 Software compilation

Vaa3D requires extensive and time-consuming compilation process. In addition to a requirement of compiling Qt4 environment, developers must construct the source code and dependent libraries. The entire compilation procedure is depicted in Figure.S1 (a). The Vaa3D native and plug-in engineering files are parsed with the qmake tool based on the development of the Qt4 component environment. The binary files are created for Visual Studio 2013 compilation, and the final link generates an executable file. The headers and links of the external dependency libraries that Vaa3D requires, however, need to be manually included by developers, which raises the configuration expenses for various development environments.

The condensed Vaa3D-x compilation procedure is illustrated in Figure.S1 (b). The framing process is made easier by the online installation of Qt6. Vaa3D-x engineering profiles (.pro) have been updated to contain paths to external dependency libraries that have been pre-processed across several platforms and saved in Vaa3D-x source code, reducing the amount of time for compilation currently.

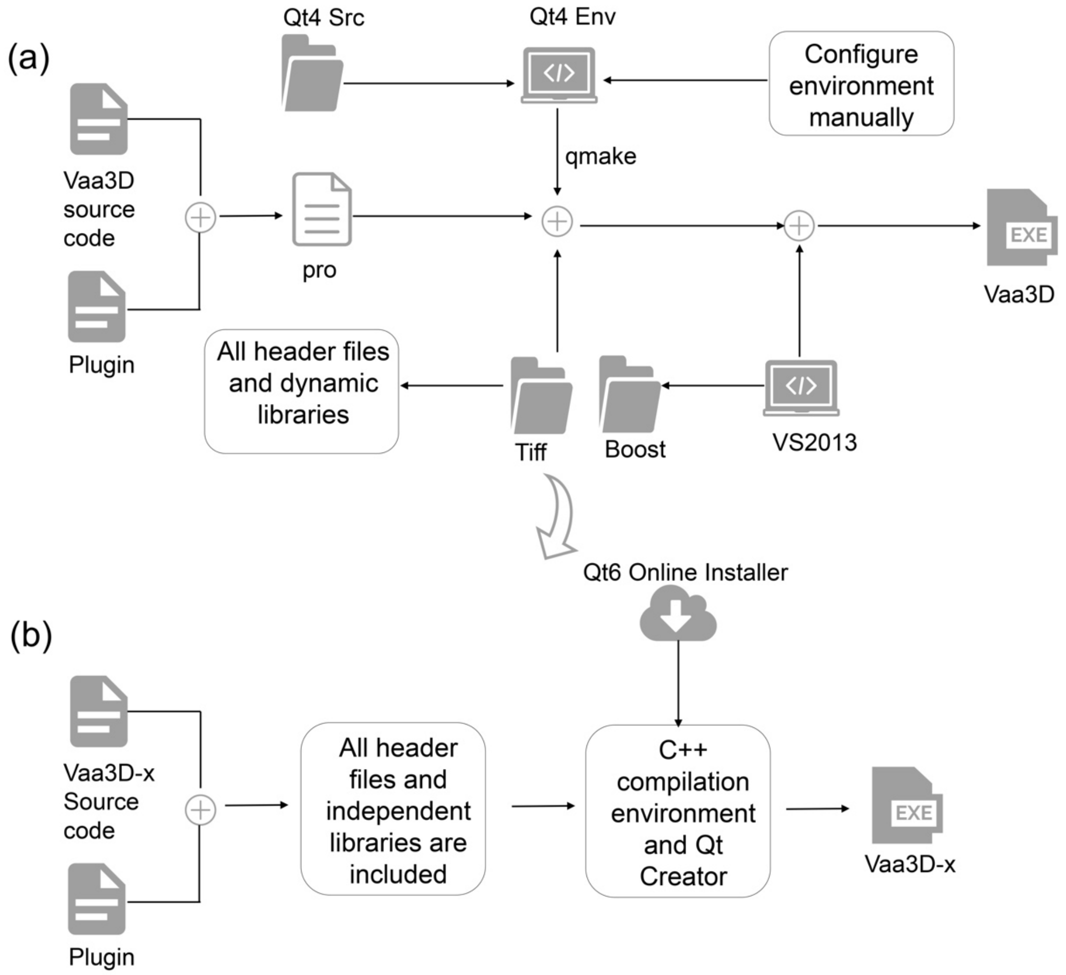

Figure.S1 The comparation of compilation process of the Vaa3D and Vaa3D-x.

3 Software Performance

3.1 Vaa3d-x Performance Test

We performed tests to illustrate the efficiency and robustness of Vaa3D-x with various 3D/4D image volumes of mouse brains. The statistics for each test case were obtained by automation script on present major system platforms. The time consumption of entering ROI image blocks in TeraFly was recorded in Figure.S2(a). The response time in Vaa3D-x TeraFly is stable within 5s, 6s, 9s in Mac, Windows and Ubuntu system respectively, no matter how the images size sharp increased. The total average time for generation, loading and displaying of a 3D viewer on three system platforms was reported in Supplementary Figure.S2(b). Remarkably, all test cases considered the hardware input and output and rendering time, the display time of Vaa3D-x was maintained at about 700ms with the image size ranging from 3.5 gigabyte to 2120 gigabyte, thus it proved stability and potential in processing terabyte multi-dimensional images.

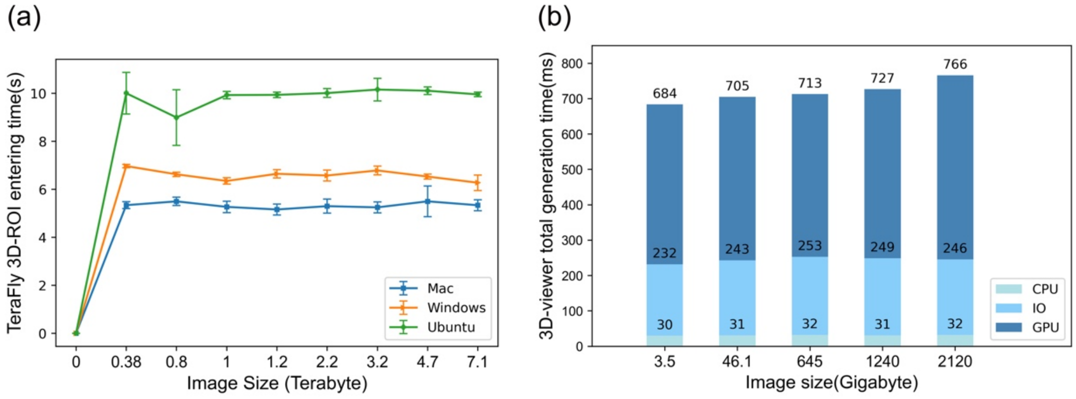

Figure.S2 illustrates the operating efficiency and robustness of Vaa3D-x. a. Time for entering ROI image blocks of eight 3D/4D image stacks with size ranging from 0.38 terabyte to 7.1 terabyte. Configuration of the tested computers: Mac, CPU Intel Core i7, RAM 16GB, GPU AMD Radeon Pro 5300M. Windows: CPU Intel i7-8700K, RAM 64gb, GTX1080. Ubuntu: Intel® Xeon® CPU E5-2650 v4, RAM 125gb, NVIDIA Corporation GP102 [Titan Xp]. **b.** Average total time for generation, loading and displaying of a 3D VOI for five 3D/4D image stacks with size ranging from 0.3 gigabyte to 2.1 terabyte. Configuration of the tested computer: Windows: Intel(R) Core (TM) i9-9900K CPU @ 3.60GHz.

3.2 Vaa3D-x visualization examples

Figure S3 shows immersive 3-D visualization, interaction, and hierarchical streaming of teravoxel-scale images in Vaa3D-x cross three platforms.

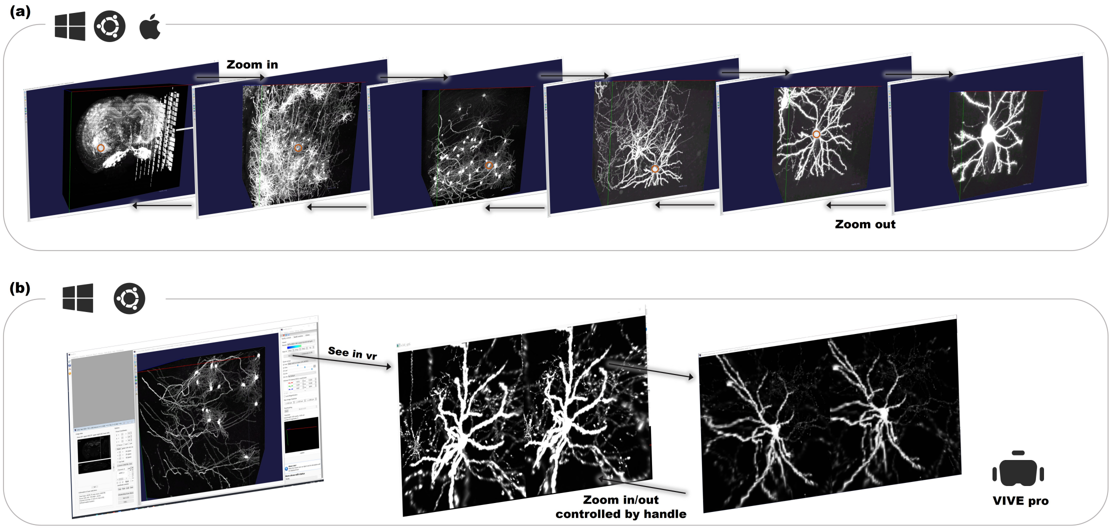

Figure.S3 Vaa3D-x visualization examples a. Cross three platforms, in a real time, users can precisely and efficiently load the data of a desired high-resolution ROI to see detailed 3-D morphological structures. **b**. Vaa3D-x can provide Windows and Ubuntu users an immersive virtual reality experience by equipping a VIVE device and pressing the 'see in vr' button on the control bar.

4 Function Testing

We have made a thorough functional testing plan for Vaa3D-x across multiple platforms and configurations to increase the confidence level of the performance of the software. We have implemented automated software testing and focused the function and performance testing in Vaa3D-x (Table.S3). We tested the main function and over one hundred plugins functions cross these three platforms (Table.S1 and Table.S4). And we also carried out a series of pressure tests to monitor the tools' response time, system limit capacity and the RAM usage in the experiments (Table.S2).

4.1 Vaa3D-x Pressure Testing

4.1.1 Test plan

Pressure testing was executed by the script based on the pyautogui library in python. The pressure testing was designed to test the performance of the software when confronted with large amounts of data and large amounts of user operations. Therefore, we imitated the manual mouse operation done in Vaa3D-x software, saved the 'click' or 'drag' image buttons in advance and recognized it through pyautogui. The mouse will be moved to the location of the image saved and did the operation of the 'click' or 'drag'. And the performance of software was recorded in Table.S2.

4.1.2 Test Environment

Table.S1 Test Environment

The composition of the system test environment	Actual Content
Server hardware	None
Client hardware	One PC
Internet connection devices	without internet access
Test instrument	None
Printer, Scanner	One printer
The software under test	Vaa3D
Operating systems	Windows 11, Ubuntu20, Mac
Database	None
Coexistence software	None
Test tool	Python script
Test data	Test Data
Related manuals	Help

4.1.3 Result

The first test is testing the maximum number of the opened images in main widget. The image size is 128*128*128*1. The response of each image is 0.2-0.5s with operation time. The RAM usage is increased with the images opening. The program performs stably when the total size of the images which are opened is less than the RAM usage. The second test is testing the maximum number of swc files in one single 3D Viewer widget. The swc file size is from 30kb to 800kb. The response time of each swc is about 0.3s when the quantity of opened swc is less than 80. The response time will slow down when the quantity of swc file is more than 80. When the quantity of swc files reach 110, the opening time can be 2s. During this test, the RAM usage can be up to 200MB, and the Mean Time Between Failures is about 20 minutes (opening the swc files by imitating manual operation).

Table.S2 Pressure Test Result

Number	Test Data	Test Content	Response time	RAM usage	Mean Time Between Failures, MTBF	Result
1	1978 Tiff files (128*128*128*1)	maximum number of opened images in main widget	0.2-0.5s	size of all the images	none	Ok
2	110 swc files	maximum number of swc files in one 3dviewer widget	0.2-2s	200mb	20min (drag the swc in 3dviewer manually)	Ok

4.2 Auto Testing of Main Function of Vaa3D-x

We tested the main functions of Vaa3D-x by writing plugins using the interface provided in the Vaa3D-x source code. The test results show that all the main functions of Vaa3D-x are working.

Table.S3 Main Function Test Result

Number	Main Function	Result
1	callback.getImageWindowList	TRUE
2	callback.currentImageWindow	TRUE
3	callback.curHiddenSelectedWindow	TRUE
4	callback.newImageWindow	TRUE

5	callback.updateImageWindow	TRUE
6	callback.getImageName	TRUE
7	callback.setImageName	TRUE
8	callback.getImage	TRUE
9	callback.setImage	TRUE
10	callback.open3DWindow	TRUE
11	callback.close3DWindow	TRUE
12	callback.openROI3DWindow	TRUE
13	callback.closeROI3DWindow	TRUE
14	callback.pushObjectIn3DWindow	TRUE
15	callback.pushImageIn3DWindow	TRUE
16	callback.closeROI3DWindow	TRUE
17	callback.getView3DControl	TRUE
18	callback.getLocalView3DControl	TRUE
19	callback.getTriviewControl	TRUE
20	callback.getVaa3DMainWindow	TRUE
21	callback.getListAll3DViewers	TRUE
22	callback.find3DViewerByName	TRUE
23	callback.getHandleNeuronTrees_3DGlobalViewer	TRUE
24	callback.getHandleAPOCellList_3DGlobalViewer	TRUE
25	callback.getListLabelSurf_3DGlobalViewer	TRUE
26	callback.setListLabelSurf_3DGlobalViewer	TRUE
27	callback.getHandleNeuronTrees_Any3DViewer	TRUE
28	callback.getHandleAPOCellList_Any3DViewer	TRUE
29	callback.getListLabelSurf_Any3DViewer	TRUE
30	callback.setListLabelSurf_Any3DViewer	TRUE
31	callback.getView3DControl_Any3DViewer	TRUE
32	callback.screenShot_Any3DViewer	TRUE
33	callback.update_3DViewer	TRUE
34	callback.update_NeuronBoundingBox	TRUE
35	callback.getHandleLandmarkList_Any3DViewer	TRUE
36	callback.setHandleLandmarkList_Any3DViewer	TRUE
37	callback.open3DViewerForSingleSurfaceFile	TRUE
38	callback.open3DViewerForLinkerFile	TRUE
39	callback.open3DViewerForLinkerFile	TRUE
40	callback.createEmpty3DViewer	TRUE
41	callback.setWindowDataTitle	TRUE
42	callback.getWindowDataTitle	TRUE
43	callback.moveWindow	TRUE
44	callback.resizeWindow	TRUE
45	callback.setHideDisplayControlButton	TRUE
46	callback.getSWCTeraFly	TRUE
47	callback.setSWCTeraFly	TRUE
48	callback.getLandmarkTeraFly	TRUE
49	callback.getPathTeraFly	TRUE
50	callback.getImageTeraFly	TRUE
51	callback.versionTeraFly	TRUE
52	callback.getSubVolumeTeraFly	TRUE
53	callback.releaseOpenedVolumesTeraFly	TRUE

54	callback.setImageTeraFly	TRUE
55	callback.setSWC_noDecompose is true	TRUE
56	callback.hideSWC	TRUE
57	callback.displaySWC	TRUE

4.3 Plugin System

We have tested every plugin's function by using auto testing script. We compared the Vaa3D and Vaa3D-x plugins function to validate whether the plugin worked well. The testing script was designed to compare the running result of three repetitions, which used the MD5 code of the tests' result. And all the plugins have worked well.

Table.S4 Plugin Test Result

Number	Test Plugins	Test Environment	Costed Times	Test Result
1	All Released plugins in Vaa3D-x	Mac: CPU<br>Intel Core i7, RAM 16GB, GPU AMD Radeon Pro 5300M	3	MD5 result is consistency
2	All Released plugins in Vaa3D-x	Windows: CPU intel i7-8700, RAM 64gb, GTX1070	3	MD5 result is consistency
3	All Released plugins in Vaa3D-x	Ubuntu: Intel® Xeon® CPU E5-2650 v4, RAM 125gb, NVIDIA Corporation GP102 [Titan Xp]	3	MD5 result is consistency

5 Testing Data

Limited by the size of test data, we provided IDs of all whole brain data as well as some additional data used to test Vaa3D-x, which is available at https://github.com/Vaa3D/Vaa3D_Data/releases/tag/data_v2.0. To obtain the full test data, please contact us.

Table.S5 IDs of all whole brain data in Figure S3.a

Data size	Data id
393G	Mouse18671_teraconvert
820G	Mouse17543_teraconvert
1014G	Mouse18462_teraconvert
1.2T	Mouse18463_teraconvert
2.2T	Mouse18458_teraconvert
3.2T	Mouse191801_teraconvert
4.7T	Mouse182725_teraconvert
7.1T	Mouse18867_teraconvert
